# Math anxiety and math motivation in online learning during stress: The role of fearful and avoidance temperament and implications for STEM education

**DOI:** 10.1371/journal.pone.0292844

**Published:** 2023-12-14

**Authors:** Danni Li, Jeffrey Liew, Dwayne Raymond, Tracy Hammond

**Affiliations:** 1 Department of Educational Psychology, Texas A&M University, College Station, Texas, United States of America; 2 Department of Philosophy, Texas A&M University, College Station, Texas, United States of America; 3 Department of Computer Science and Engineering, Texas A&M University, College Station, Texas, United States of America; St John’s University, UNITED STATES

## Abstract

Students’ math motivation can predict engagement, achievement, and career interest in science, technology, engineering, and mathematics (STEM). However, it is not well understood how personality traits and math anxiety may be linked to different types or qualities of math motivation, particularly during high-stress times such as the COVID-19 pandemic. In this study, we examined how fearful or avoidant temperaments contribute to math anxiety and math motivations for college students during the COVID-19 pandemic. Ninety-six undergraduate students from a large public university were assessed on temperamental fear, math anxiety, and math motivation in an online math course. Results showed that higher levels of temperamental fear are directly linked to higher levels of math anxiety. In addition, temperamental fear is indirectly linked to higher levels of autonomous motivation (i.e., intrinsic motivation and identified regulation) and lower levels of controlled motivation (i.e., external regulation) through math anxiety. Results have implications for helping students at high risk for both high math anxiety and for low motivation to engage in math learning.

## Introduction

Primary, secondary, and tertiary or higher education programs prioritize science, technology, engineering, and mathematics (STEM) education because STEM-related skills support economic development and address global challenges [1]. The labor market and employment trends for talented individuals with strong STEM-related skills are evident. However, less is known about individual differences in personality and affective and attitudinal states that impact motivation to engage in STEM learning, particularly in online learning. Technical challenges and limited support can pose difficulties for certain students in adapting to online learning [2]. Indeed, the rapid shift to online learning during the COVID-19 pandemic significantly increased academic stress for college students [3]. Furthermore, in developed Western countries, students’ motivation to engage in STEM education has declined in the past two decades [4]. This decline is concerning given the evidence linking students’ STEM motivation, achievement in STEM-related courses, and STEM career choices [5, 6]. Considering the high-stakes and increased prevalence of online learning, the present study aimed to understand how college students’ personality styles and affective responses to mathematics contribute to the type or quality of their mathematics motivation. Specifically, the present study examined the processes by which college students’ fearful temperament traits contribute to math anxiety and math motivation in an online math course during the coronavirus disease 2019 (COVID-19) global pandemic, a high-stress time for students. During the COVID-19 pandemic, most college students reported increased stress level due to academic-, health-, and lifestyle-related concerns caused by the pandemic [3, 7].

**Fig 1:**
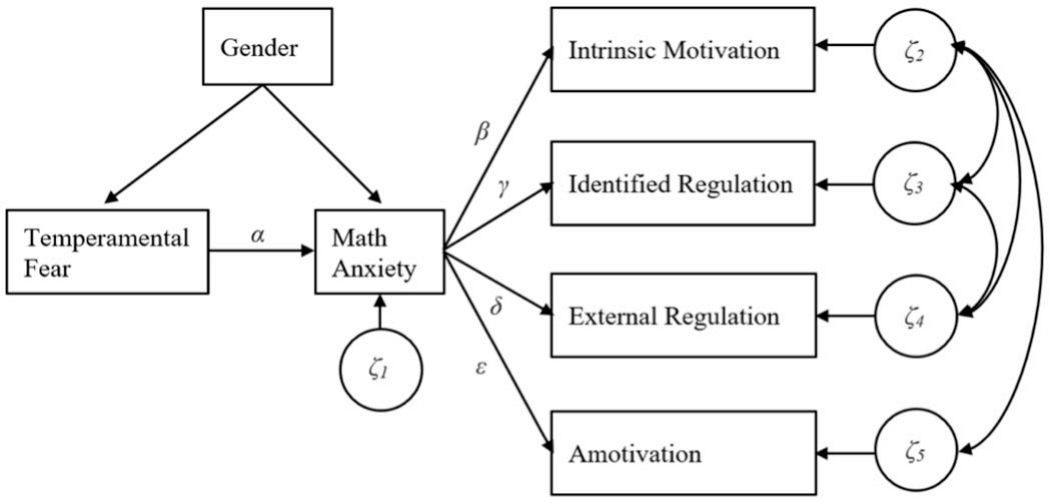
Hypothesized indirect effect model.

Math anxiety is defined as the physiological tension, worrying feeling, and avoidance behavior when facing math-related stimuli [8]. Although research has established that math anxiety is associated with reduced motivation in general toward mathematics [9, 10], whether and how math anxiety may be linked to different types of math motivation is not well understood [11]. Further, these relationships need to be further explored in the context of online learning environments and high-stress times. Dowker et al. called for more research on how math anxiety relates to different aspects of motivation [12]. Furthermore, when learning occurs primarily online during high-stress times, like the COVID-19 pandemic, students’ learning and academic performance may be severely impaired [13]. By understanding links between temperamental fear, math anxiety, and different types of math motivation, we could identify students prone to underperforming in mathematics. Likewise, we could identify entry points, interventions, or supports that effectively enhance students’ mathematics motivation and learning. The present study aimed to examine the roles of temperamental fear and math anxiety in four types of motivations (i.e., intrinsic motivation, identified motivation, external motivation, and amotivation) among undergraduate students in online learning settings. Specifically, we tested the indirect effects of temperamental fear on different types of math motivation through math anxiety (see Fig 1).

### Amotivation, controlled motivation, and autonomous motivation

Motivation has been viewed as a multidimensional affective construct. According to self-determination theory (SDT) [14], autonomous motivation, controlled motivation, and amotivation exist on a continuum from high to low levels of autonomy (i.e., self-determination), respectively. Specifically, *autonomous motivation* occurs when people choose to do an activity of their own volition. Ryan and Deci proposed several types of autonomous motivation [14]. For example, *intrinsic motivation* refers to choosing to engage in an activity because it is inherently pleasurable and satisfying. Likewise, *identified regulation* refers to choosing to do an activity because one perceives it as important or useful. In contrast, *controlled motivation* occurs when people engage in an activity due to external pressure. A type of controlled motivation is *external regulation*, which refers to the motivation to achieve rewards or avoid negative consequences associated with the activity. Lastly, *amotivation* refers to the state of lacking either autonomous or controlled motivation and lacking a sense of purpose or expectations.

Importantly, these motivation types are differently related to various outcomes. According to SDT, autonomous motivation (i.e., intrinsic motivation and identified regulation) improves performance and outcomes—such as cognitive performance [15], academic achievement [16–18], and psychological well-being [19]—because individuals engage in the activity due to their interests and desires. In contrast, when driven by external rewards or the approval of others, as with controlled motivation (i.e., external regulation), individuals’ performance and learning outcomes are expected to be worse or unreliable. However, findings are mixed on the associations between controlled motivation and performance or academic outcomes. Some studies show that controlled motivation predicts poor academic outcomes [20], whereas others show no relationship [21]. Finally, amotivation has been linked to poor achievement and academic outcomes. In one study, academic amotivation predicted lower academic achievement and higher school dropout rates among high school students [22]. According to Ryan and Deci [23], individuals tend to show less autonomous motivation and more controlled motivation when their basic psychological needs are not met, such as when experiencing high levels of math anxiety. In the present study, we hypothesize that math anxiety is associated with lower levels of autonomous motivation (i.e., intrinsic motivation and identified regulation) and higher levels of controlled motivation (i.e., external regulation) and amotivation.

### Math anxiety

Researchers have identified complex interplay between emotion and cognition in math learning, with math anxiety being a critical affective factor in motivation and learning of mathematics [11]. Although math anxiety closely relates to test anxiety and general anxiety (*r* = 0.3–0.5), it is a separate concept because different measures of math anxiety are highly correlated (*r* = 0.5–0.8) [12, 24]. Female students report significantly higher levels of math anxiety compared to male students [25]. Studies have shown that math anxiety is associated with compromised working memory and poor math achievement [26, 27]. Although students with higher math anxiety report lower math motivation in general [8, 28], recent studies challenge the linear relationship between math anxiety and motivation. One study, for example, found that some students with higher math anxiety might be highly motivated in mathematics [29]. Most previous studies examined math motivation as a general structure [2, 28], but few studies examined the relationship between dimensions of motivation in the SDT framework and math anxiety. Studies that have examined this relationship report mixed findings. For example, some studies found that math anxiety and intrinsic motivation are negatively correlated [11], whereas other studies found no significant correlation [30]. In the present study, we aim to elucidate the relationship between math anxiety and motivation in the SDT framework. Further, we test the indirect effects of temperamental fear on four types of math motivation (i.e., intrinsic motivation, identified motivation, external motivation, and amotivation) in the context of a high-stress time—the COVID-19 pandemic.

### Temperamental fear

Fear is a basic or primary negative emotion stemming from the perception of psychological or physical threat [31]. Thus, fear arises in stressful and unpredictable events, such as the COVID-19 pandemic. Temperament in adults refers to the personality traits individuals bring to various contexts that can dictate how an individual acts or reacts. Female students show significantly higher levels of temperamental fear compared to their male counterparts [32–34]. Temperamental fear can be viewed as a subdomain of *avoidance temperament*, which represents the biological sensitivities to punishment stimuli in reality or imagination [35]. Avoidance temperament predisposes people to experience anxiety and fear in stressful situations, such as the COVID-19 pandemic. For example, Liew et al. demonstrated the impacts of avoidance temperament (i.e., temperamental fear) on college students’ test anxiety and math performance, wherein students with high temperamental fear or avoidance temperament were more vulnerable to experiencing text anxiety and performed worse in math [36]. Similarly, students with high temperamental fear can experience more anxiety toward math-related activities, which may then impact their math motivation toward math courses. According to findings from Ma’s meta-analysis on math anxiety and math achievement, math anxiety is moderately correlated with trait anxiety [37]. However, few studies have examined the relationship between temperamental fear and math anxiety, particularly in the context of heightened and chronic stress, such as that of a global pandemic.

Furthermore, math anxiety relates to general anxiety [38]. Because anxiety is a secondary emotion, it emerges from or is experienced after a basic or primary emotion. For example, general or domain-specific anxiety (e.g., math anxiety) can serve as a secondary emotion to the primary emotion of fear. Stressful situations, such as the COVID-19 pandemic and the new form of online learning, could increase the anxiety level of students, and we expect that math anxiety would also increase in this context. Therefore, in the present study, we hypothesize that temperamental fear predicts high levels of math anxiety.

### Present study

This study took place during the COVID-19 pandemic, which abruptly forced educational institutions worldwide to temporarily close and transition from in-person instruction to online learning environments. The present study aimed to examine the relationship between college students’ temperamental fear, math anxiety, and motivation in online learning during a high-stress time. Specifically, we anticipate a positive impact of temperamental fear on math anxiety. In addition, we hypothesize that higher temperamental fear indirectly affects four types of math motivation (i.e., lower levels of intrinsic motivation, lower levels of identified motivation, higher levels of external motivation, and higher levels of amotivation) through math anxiety.

## Methods

### Participants

Participants were undergraduate students at a large public university in the southwestern United States who enrolled in a university-level math course in the 2020 fall semester with fully online instruction due to the COVID-19 pandemic. Of the 121 undergraduate students who participated in the study, 96 students (79.3%) finished the online survey. The demographic characteristics of participants are presented in Table 1. Among the 96 participants, 47 (49.0%) were male. The majority of participants (52.1%) identified as Non-Hispanic White, followed by 27.0% Hispanic American, 8.3% Asian American, 6.3% African American, and 6.3% Other. In addition, 42.7% of participants were sophomores.

**Table 1:** Characteristics of participants (N = 96).

		N	%
Gender	Male	47	49.0%
	Female	49	51.0%
Ethnicity	African American	6	6.3%
	Asian American	8	8.3%
	Hispanic American	26	27.0%
	Non-Hispanic White	50	52.1%
	Other	6	6.3%
Year	Freshman	17	17.7%
	Sophomore	41	42.7%
	Junior	22	22.9%
	Senior	16	16.7%

### Procedure

The study was approved by the university’s Institutional Review Board (IRB). Data were collected from students in a university-level math course using an anonymous online survey. The instructor of the math course invited students to participate in this study in August 2020 by sharing the study information and a web link to the anonymous online survey. Participation was strictly voluntary, and participants gave written consent electronically after being presented with study information. Participants then continued to the next computer screen to complete the survey, reaffirming their consent to take part in the study.

### Measures

#### Temperamental fear

Temperamental fear was measured by seven items (e.g., “Sometimes, I feel a sense of panic or terror for no apparent reason.”) from the Adult Temperament Questionnaire–Short Form (ATQ) [31]. The ATQ comprises 100 items and uses a 7-point Likert-type scale (i.e., 1 = *extremely untrue* to 7 = *extremely true*). In our study, the internal consistency of the measurement is good (Cronbach’s alpha = .75).

#### Math anxiety

Math anxiety was measured by the Abbreviated Math Anxiety Scale (AMAS) [39]. The AMAS contains 9 items and uses a 5-point Likert-type scale (i.e., 1 = *low anxiety* to 5 = *high anxiety*). Each item describes a situation related to math study or testing (e.g., “Thinking about an upcoming math test one day before”), and participants reported their anxiety level during each event. In our study, the coefficient alpha of the AMAS is .90.

#### Math motivation

Participants reported on their math motivation using an adapted version of the Situational Motivation Scale (SIMS) [40], a 16-item instrument designed to measure motivation during an activity. Each item is rated on a 7-point Likert-type scale (i.e., 1 = *not at all* to 7 = *exactly*). The SIMS items were modified to measure participants’ motivation to take the math course. The participants were asked, “Why are you currently engaged in this [math] class?,” and each item described a potential reason (e.g., “Because it is something that I have to do.”). The SIMS consists of four subscales: intrinsic motivation, identified regulation, external regulation, and amotivation, with each subscale being measured by 4 items. For our sample, the coefficient alpha is .91 for the intrinsic motivation subscale, .83 for the identified regulation subscale, .78 for the external regulation subscale, and .81 for the amotivation subscale.

### Statistical analysis

The data were prepared and analyzed using Stata 16. Of the 121 students who started the online survey, 25 students did not complete it. Using Little’s Missing Completely at Random (MCAR) test, the missingness was determined completely random. Thus, listwise deletion of these 25 cases was conducted [41]. Descriptive statistics and binary correlations were then examined. The hypothesized path model was analyzed with the maximum likelihood (ML) estimation method. The model was evaluated with overall model chi-square test and other goodness of fit statistics (i.e., CFI, RMSEA, SRMR) [42]. To examine the four indirect effects (αβ, αγ, αδ and αε), a bootstrapping method with the computation of the 95% confidence intervals was used [43].

## Results

### Descriptive statistics

Descriptive statistics and the binary correlations of study variables are presented in Table 2. Female students reported significantly higher levels of temperamental fear and math anxiety. As hypothesized, temperamental fear was significantly positively correlated with math anxiety. Math anxiety was negatively correlated with intrinsic motivation and identified regulation. In contrast, math anxiety was positively correlated with external regulation and amotivation. Additionally, the four types of motivations correlated in the expected directions with each other, excluding external regulation and amotivation. Independent samples t-tests were conducted to examine potential gender differences for study variables. The results showed that female students (*M* = 4.47, *SD* = 1.07) reported significantly higher levels of temperamental fear than male students (*M* = 3.09, *SD* = 1.16); *t*(94) = -6.07, *p* < .001. Similarly, female students (*M* = 3.08, *SD* = .88) reported significantly higher levels of math anxiety than male students (*M* = 2.27, *SD* = .72); *t*(94) = -4.95, *p* < .001. No significant gender differences were found among motivation variables.

**Table 2:** Descriptive statistics and binary correlations (N = 96).

		1	2	3	4	5	6	7
1.	Gender^a^	-						
2.	Temperamental Fear	.53**	-					
3.	Math Anxiety	.45**	.48**	-				
4.	Intrinsic Motivation	-.03	-.10	-.41**	-			
5.	Identified Regulation	-.07	-.17	-.49**	.78**	-		
6.	External Regulation	-.03	.01	.25*	-.33**	-.32**	-	
7.	Amotivation	-.02	.04	.25*	-.40**	-.56**	.16	-
	Mean	-	3.79	2.68	3.18	3.93	4.80	3.17
	Standard Deviation	-	1.31	.90	1.29	1.40	1.44	1.31

^*a*^ For coding of gender, males = 1 and females = 2.

*p < .05,

**p < .01.

### Path model and tests of indirect effects

The hypothesized indirect effect model is presented in Fig 1. All variables in the model are observed variables. In the model, temperamental fear was hypothesized to be directly linked to math anxiety (path *α*). In addition, temperamental fear was hypothesized to be indirectly linked through math anxiety to intrinsic motivation (path *β*), identified regulation (path *γ*), external regulation (path *δ*), and amotivation (path *ε*), respectively. To account for gender differences discovered through the independent samples t-tests, gender was included as a covariate for temperamental fear and math anxiety.

Table 3 presents path model results with standardized coefficients and model fit indexes. A chi-square difference test and model fit indexes showed that the hypothesized model was a good fit for the data: *χ*^2^(9) = 8.02, *p* = .532, CFI = 1.00, RMSEA = .00, and SRMR = .07, respectively. The cut-offs of the indexes for a good fit are *p* > .05, CFI ≥ .90, RMSEA < .08, and SRMR < .08 [44]. After controlling for gender effects, the direct path between temperamental fear and math anxiety was significant ($\hat{\alpha }=.33$, *p* = .001). In addition, the three direct paths from math anxiety and low intrinsic motivation ($\hat{\beta }=-.41$, *p* = .000) were all significant: (1) low identified regulation ($\hat{\gamma }=-.49$, *p* = .000), (2) high external regulation ($\hat{\delta }=.25$, *p* = .008), and (3) high amotivation ($\hat{\varepsilon }=.25$, *p* = .010). Furthermore, the three indirect effects estimates ($\hat{\alpha \beta },\;\hat{\alpha \gamma }$, and $\hat{\alpha \delta }$) were statistically significant given that the corresponding bootstrap 95% confidence intervals did not include zero ($\hat{\alpha \beta }=-.14$, 95% CI = [−0.237, −0.033]; $\hat{\alpha \gamma }=-.17$, 95% CI = [−0.292, −0.056]; and $\hat{\alpha \delta }=.09$, 95% CI = [0.005, 0.181], respectively). Such results indicate that, through math anxiety, temperamental fear has significant indirect effects on three of the four types of motivations (i.e., intrinsic motivation, identified regulation, and external regulation). However, the indirect effect between temperamental fear and amotivation was not significant ($\hat{\alpha \varepsilon }=.08$, 95% CI = [-0.003, 0.167]). Path model results indicate that, even after controlling for gender, participants with higher levels of temperamental fear were more likely to report experiencing higher levels of math anxiety. In addition, higher levels of math anxiety then linked to lower levels of intrinsic motivation and identified regulation as well as higher levels of external regulation.

**Table 3:** Standardized parameter estimates for the study (N = 96).

	Estimates (SE)		*Z* value
*Path coefficients*		*p*	
Gender → Temperamental Fear	.53 (.07)	.000	7.83
Gender → Math Anxiety	.28 (.10)	.005	2.81
*Direct effects*		*p*	
*α*	.33 (.10)	.001	3.41
*β*	-.41 (.08)	.000	-4.88
*γ*	-.49 (.08)	.000	-6.38
*δ*	.25 (.10)	.008	2.66
*ε*	.25 (.10)	.010	2.56
*Indirect effects* ^a^			
*αβ*	-.14	95% CI^b^ = [−0.237, −0.033], Reject *H*_*0*_^c^	
*αγ*	-.17	95% CI = [−0.292, −0.056], Reject *H*_*0*_	
*αδ*	.09	95% CI = [0.005, 0.181], Reject *H*_*0*_	
*αε*	.08	95% CI = [-0.003, 0.167], Support *H*_*0*_	
*Model fit indexes*			
Chi-square (df)	8.02 (9)	*p* = .532	
CFI	1.00		
RMSEA	.00		
SRMR	.07		

^*a*^Based on the unstandardized coefficients; the standard errors of *αβ*, *αγ*, *αδ* and *αε* were not estimated due to the use of bootstrapping methods.

^*b*^CI: Confidence interval.

^*c*^*H*_*0*_: The null hypothesis of the indirect effect, which was set to equal zero.

## Discussion

Study results confirmed that temperamental fear indirectly affect different types of academic motivation through math motivation among college students engaged in online learning during a high-stress time. Specifically, higher levels of temperamental fear were directly linked to higher levels of math anxiety, which then were linked to higher levels of autonomous motivation (i.e., intrinsic motivation and identified regulation) and lower levels of controlled motivation (i.e., external regulation). Study results supported all but one of our hypotheses; that is, the indirect effects of math anxiety were significant between temperamental fear and all types of motivation except for amotivation. Because amotivation is a state of motivational apathy, it may not be linked to or driven by anxiety. Rather, amotivation or apathy in math can be partly driven by pessimistic beliefs about math ability, a lack of interest in exerting effort in math, or a lack of interest in math as a subject. Thus, students who exhibit amotivation in math may benefit from different intervention strategies than students who exhibit controlled or external motivation. Overall, our study findings could inform diagnosing and designing learning environments, curriculum, and teacher practices that support students. Through such practices, students’ psychological needs of autonomy, competence and relatedness could be met, promoting self-determined types of motivation for math learning and performance.

### Temperamental fear, math anxiety, and math motivation

The present study examined the indirect effect of temperamental fear on math motivation through math anxiety in an online college math course during a high-stress time for students (i.e., the COVID-19 pandemic). Similar to Ma’s [37] finding that trait anxiety is moderately correlated with math anxiety, we found that temperamental fear is positively correlated with math anxiety. As a more distal predictor of motivational processes [45], temperamental fear was not directly related to any of the four types of math motivation. Consistent with research that avoidance temperament and temperamental fear may predispose individuals to distress and anxiety in stressful situations [35, 46], students with higher levels of temperamental fear may experience more math anxiety. This relationship may be particularly evident when learning environments abruptly shift from in-person to online during a high-stress global pandemic, which would decrease their autonomous motivation and increase controlled motivation. Importantly, autonomous motivation predicts enhanced academic outcomes [15, 16, 19], whereas controlled motivation predicts poor academic outcomes [20]. Our results suggest that if math anxiety can be reduced, we can expect increased autonomous motivation and reduced controlled motivation for math learning, even if the students have high levels of temperamental fear that might predispose them to experiencing math anxiety. Understanding this relationship is particularly salient in online learning environments and during high-stress times for students, such as the COVID-19 pandemic, wherein instructors can support STEM learning for students who may be especially vulnerable to math anxiety and losing motivation to engage in math learning.

### Gender gap in math anxiety

Consistent with previous research [47], female students reported significantly higher levels of temperamental fear than male students. Similarly, female students reported experiencing higher math anxiety than male students. No significant gender differences were found in math motivation. Previous studies have found a gender difference in self-reported math anxiety [32–34]. However, Flessati and Jamieson [47] attribute the difference to response bias because females are more self-critical of math anxiety and math performance than males. Moreover, Goetz et al. [48] found that although female students reported higher levels of math anxiety than male students, no gender differences were found in state anxiety during a math test. Goetz et al. [48] also argued that female students reported higher levels of math anxiety because they reported lower perceived math competence than male students, even without significant gender differences in math grades. Thus, the present study contributes to the literature by documenting temperamental fear as a possible explanation for gender differences observed in math anxiety.

### Limitations and future directions

The present study illuminates the mechanism by which temperamental fear and math anxiety may impact different types of math motivation. Study findings should be interpreted with several study limitations in mind. Regarding the research sample, sample size and characteristics may limit the generalizability of the study. According to the N:q rule by Jackson [49], an ideal sample-size-to-parameters ratio for SEM is 20:1 and an acceptable ratio is 10:1. For the current study, the N:q ratio is 16:1, which falls between acceptable and ideal. Additionally, all participants were recruited from the same class, and most students were Non-Hispanic White or Hispanic. Our findings must be replicated with large diverse samples as well as in online learning environments with varying levels of acute and chronic stress. Likewise, because the present study uses cross-sectional data, longitudinal studies are needed to establish a causal relationship in future studies. Nonetheless, all measures demonstrate good psychometric properties, including reliability, and demonstrate validity in prior published studies.

In conclusion, this study demonstrates that, through math anxiety, temperamental fear indirectly affects multiple types of math motivation in an online math course during a high-stress time. Study findings highlight that some students are more vulnerable to experiencing math anxiety and losing motivation to engage in math learning, particularly in online learning environments and high-stress times. Such outcomes could place these students at risk for underperformance or failure in math and school in general. For students at high risk for math anxiety and losing motivation to engage in math learning, evidence-based interventions can reduce students’ math anxiety and increase autonomous motivation [50, 51]. For example, expressively writing about worries before taking math exams or tasks can significantly improve math performance, especially for students with high levels of math anxiety [27, 52]. Additionally, to reduce math anxiety for online math courses, teachers can diversify teaching styles with online resources, separate the material into smaller chunks, and create a more positive learning environment in which students feel comfortable asking questions [53]. Ultimately, the findings of this study underscore the need for educators, parents, and policymakers to recognize the complex interplay between temperamental fear, math anxiety, and motivation, and to implement the suggested interventions proactively to foster a supportive and low-anxiety learning environment, particularly in online settings where stress may be exacerbated.

## Supporting information

S1 Dataset (XLSX)
